# Indium-mediated C-allylation of melibiose

**DOI:** 10.3762/bjoc.15.238

**Published:** 2019-10-16

**Authors:** Christian Denner, Manuel Gintner, Hanspeter Kählig, Walther Schmid

**Affiliations:** 1 Institute of Organic Chemistry, University of Vienna, Währinger Straße 38, A-1090, Vienna, Austria

**Keywords:** carbohydrates, C–C bond formation, indium-mediated allylation, melibiose, ozonolysis

## Abstract

The indium-mediated allylation reaction has been applied to melibiose, a disaccharidic substrate. This elongation methodology allows for a short, efficient and diastereoselective approach towards complex glycosylated carbohydrate structures. The stereochemical outcome of the key intermediates, allylated disaccharides, has been determined by X-ray analysis. Ozonolysis of the introduced double bond yielded the unprotected elongated disaccharides in the equilibrium of the pyranoid as well as furanoid isomers in both anomeric forms, respectively. Per-*O*-acetylation has been performed to facilitate separation of the isomeric mixture for structural identification. The main product revealed to adopt a β-pyranoid form of the elongated unit at the reducing end of the disaccharide.

## Introduction

The tin and indium-mediated allylation (IMA) proved to be useful synthetic tools for the chain elongation of unprotected carbohydrates at the anomeric position to obtain higher complex sugar structures. Prior to the establishment of these methods, synthetic approaches towards these compounds had to be performed on protected carbohydrates, increasing the number of synthetic steps intrinsically [1]. In 1991, Schmid and Whitesides reported for the first time a tin-mediated allylation of unprotected carbohydrates followed by ozonolysis allowing for easy accessibility of the corresponding elongated sugars [2]. In the same year, Chan and Li introduced indium for the allylation of aldehydes and furthermore demonstrated the applicability of this post-transition metal for the elongation of carbohydrates [3–5]. Additional contributions were reported in the literature by Paquette and co-workers concerning indium-mediated reactions in water and their stereochemistry [6–7]. Based on these findings this elongation method has been established as reliable and efficient approach in carbohydrate chemistry, shown by many examples reported in the literature [8–12]. Thus far, this method has been applied to monosaccharides [13–14]. In this context, the development of diastereoselective and efficient synthetic routes to elongated disaccharides employing glycosylated substrates has been the focus of our interest. The demand for such elaborate compounds is undoubted and continuously increasing, constituting an important research aim in glycosciences. Herein, we describe the optimization of reaction conditions and structural elucidation of compounds derived from the indium-mediated C-allylation reaction employing melibiose (**1**) as disaccharidic substrate.

## Results and Discussion

**Scheme 1:**
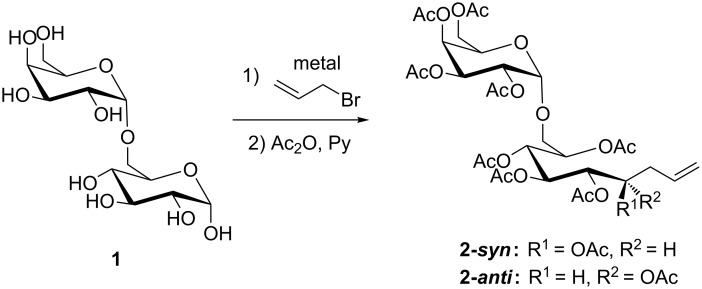
Indium-mediated allylation of melibiose (**1**).

We employed melibiose (**1**) as model substrate for the indium-mediated allylation reaction. In this disaccharide the glycosidic linkage is present at position O-6 of the glucose moiety allowing most spatial freedom for the elongation reaction at the anomeric position of the reducing end of the disaccharide (Scheme 1). First, we investigated different reaction parameters such as the metal species, the solvent system, the activation by sonication as well as the temperature concerning their effects on the conversion rate as well as the diastereoselectivity (Table 1).

**Table 1:** Optimization of the reaction conditions of the indium-mediated allylation of melibiose.

Entry	Metal	Solvent^a^	Method	Time (h)	Conversion (%)^b^

1	Sn	4:1	sonication	24	19
2	In	1:1	sonication	24	44
3	In	4:1	sonication	24	56
4	In	9:1	sonication	24	45
5	In	4:1	sonication	46	78
6	In	4:1	65 °C	7	88
7^c^	In	4:1	65 °C	4	98

^a^General conditions: 200 mg (0.577 mmol) of melibiose (**1**), 4 equiv of indium and 6 equiv of allyl bromide in 20 mL of the solvent mixture ethanol/water. ^b^Determined by NMR spectroscopy. ^c^Vigorous stirring.

Concerning the metal species, it is known that indium provides several advantages compared to other allylation-mediating metals such as tin, as it allows the reaction to proceed under very mild conditions without the need for acid catalysis or other promotors [15]. This was also observed in our studies reflected in the significantly higher conversion rates employing indium versus tin (Table 1, entries 1 and 3).

With respect to the solvent system, different ratios of an ethanol/water mixture were investigated showing that these have only minor effects on the conversion. However, the best results were obtained with a ratio of 4:1 (v/v) ethanol/water (Table 1, entries 2–4).

Although activation via sonication proved to be beneficial in our previous studies in the C-allylation of monosaccharides [16], in this case this form of energy input itself turned out to be insufficient. The best results were obtained by heating the reaction mixture with a conventional oil bath under vigorous stirring. This can be rationalized by the lower spacial availability at the reducing end of disaccharides compared to simple monosaccharides. Temperatures up to 65 °C led to elevated reaction rates (Table 1, entries 3, 5 and 6). However, higher temperatures caused concentration phenomena, which derived from the reflux of ethanol leading to precipitation of the starting material, which resulted in a significant decrease of the reaction rate. Additionally, intense stirring of the reaction mixture thereby preventing indium powder cluster formation (Table 1, entry 7) turned out to be of advantage for the progress of the reaction.

**Scheme 2:**
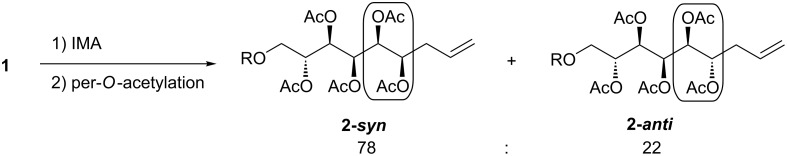
Diastereomeric ratio of allylation; R = per-*O*-Ac-α-Gal.

After the allylation subsequent per-*O*-acetylation gave a mixture of respective epimeric products at position C-4, **2-*syn*** and **2-*anti***, respectively, as well as the α,β-mixture of unreacted melibiose (**1**). Separation of the reaction mixture could be achieved by silica gel column chromatography. The diastereomeric ratio of 78:22 (**2-*syn***/**2-*anti***) remained unchanged throughout the different conditions of optimization procedures (Scheme 2).

**Figure 1:**
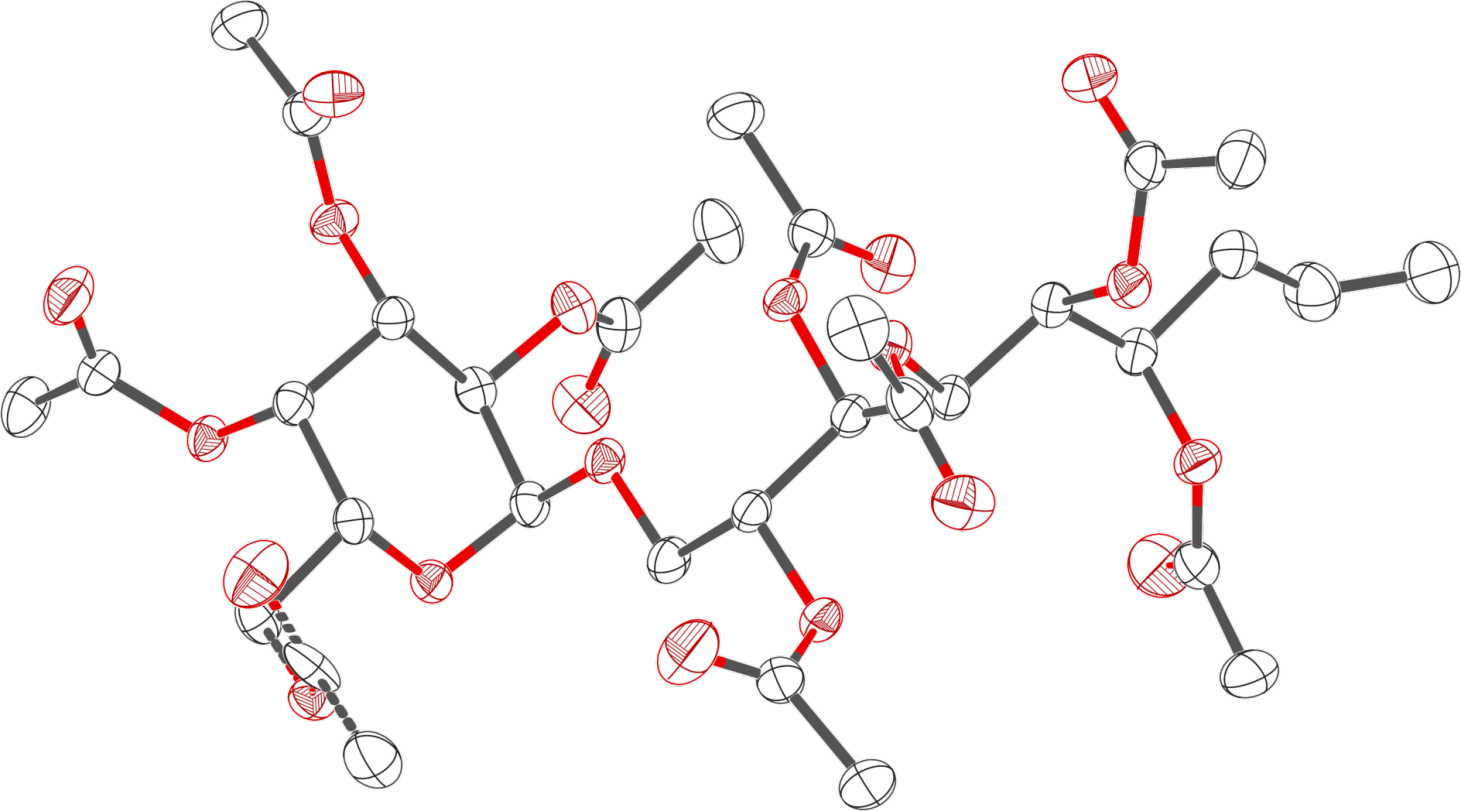
X-ray analysis of the main C-allylation product **2-*syn*** [CCDC 1922520].

The overall yield of the isolated epimers was 92%. According to Binder et al. [17] and our own experience [18], we expected the *syn*-product (**2-*syn***) to be the main diastereomer. However, the determination of the configuration is not possible by NMR analysis. Consequently, the main product was crystallized and the configuration was determined by X-ray analysis proving the expected *syn*-configuration (compound **2-*syn***) [CCDC 1922520] (Figure 1).

**Scheme 3:**
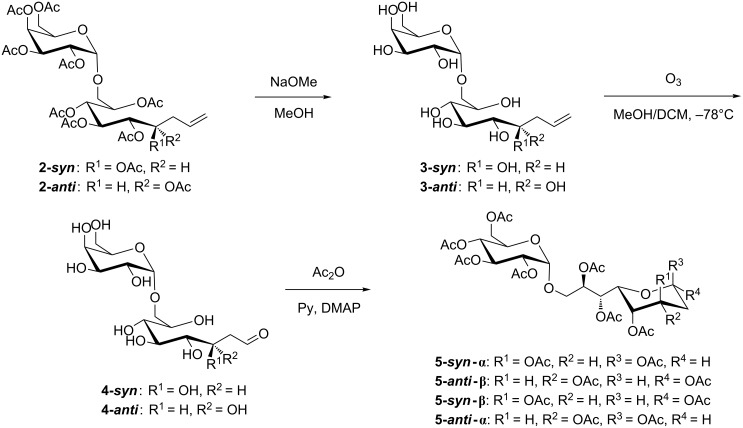
Reaction scheme of the ozonolysis sequence.

Epimer **2-*syn*** was applied to Zemplén conditions prior to the ozonolysis. Conventional work-up, neutralization with acidic ion exchange resin followed by filtration and removal of the solvent under reduced pressure, led to precipitation of compound **3-*syn***, thereby causing problems during the ozonolysis. Thus, dichloromethane was added to the reaction mixture immediately after completed Zemplén-deprotection (as indicated by TLC) without further work up. This reaction mixture was cooled to −78 °C, purged with ozone for two minutes followed by reductive work-up employing triphenylphosphine which gave the corresponding glycosylated elongated aldoses **4-*syn*** in full conversion as determined by TLC. Epimer **2-*anti*** was reacted accordingly, after Zemplén-deprotection and ozonolysis compound **4-*anti*** was obtained. Purification at this stage utilizing conventional column chromatography was not feasible due to the high polarity of the obtained compound mixture, which contained pyranoid as well as furanoid isomers in their α,β-forms. Thus, per-*O*-acetylation was conducted leading to a mixture of all four species, indicated by NMR analysis (Scheme 3).

**Scheme 4:**
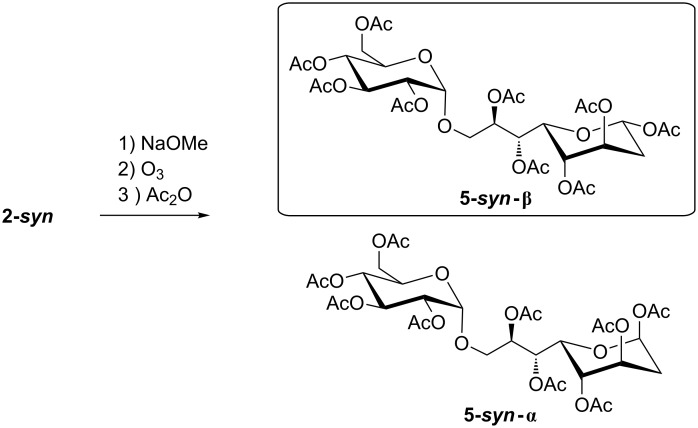
Ozonolysis sequence for the *syn*-product.

In the case of **2-*syn*** the β-pyranose species **5-*syn*-β** was obtained as the main product, besides α-pyranose (**5-*syn*-α**) as well as both anomers of the furanoid form (Scheme 4). The overall yield obtained over three steps was 72% (4 species), from this mixture the main product **5-*syn*-β** was isolated in 58% yield and could be fully characterized by NMR (Supporting Information File 1).

**Figure 2:**
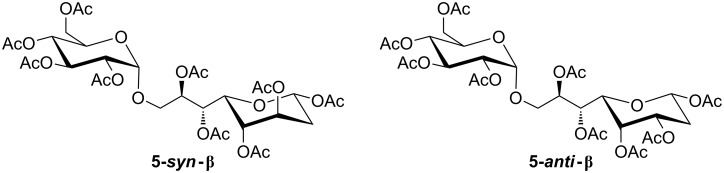
Structures of the main products **5-*syn*-β** and **5-*anti*-β**.

In the case of **2-*anti*** the same reaction sequence gave an overall yield of 65%, which is a slightly lower conversion compared to **2-*syn***. However, the main species, **5-*anti*-β** was obtained in 60% from this mixture (Figure 2).

## Conclusion

For the first time, an indium-mediated allylation reaction was employed with a disaccharide, melibiose (Gal*p*α1-6Glc, **1**), as substrate, achieving good diastereoselectivity and excellent yields. Best conversion (98%) was obtained by employing indium as the metal species, a solvent mixture of ethanol/water 4:1 (v/v), at a temperature of 65 °C under vigorous stirring. The resulting epimeric forms were isolated and the stereochemical outcome was determined via X-ray structure analysis of the per-*O*-acetylated species, **2-*syn***/**2-*anti*** 78:22. Subsequent ozonolysis applied on deprotected elongated compounds **3** generated the respective glycosylated aldoses in an anomeric mixture of both, pyranoid and furanoid forms. Per-*O*-acetylation of this mixture facilitated purification by silica gel chromatography. The main product turned out to adopt the β-pyranoid form, **5-*syn*-β** and **5-*anti*-β**, respectively, determined by thorough NMR analysis, in an overall yield of 40% and 42% over three steps calculated from epimers **2-*syn*** and **2-*anti*,** respectively. This work demonstrates the indium-mediated allylation reaction to be a powerful synthetic tool for the efficient and diastereoselective elongation of melibiose (**1**) without affecting the existing glycosidic linkage, allowing for additional options for further modifications of complex glycosides.

## Experimental

**2’,3’,4’,6’-Tetra-*O*-acetyl-α-ᴅ-galactopyranosyl-(1’→1)-2,3,4,5,6-penta-*O*-acetyl-7,8,9-trideoxy-ᴅ-*glycero*-ʟ-*gulo*-8,9-nonenitol (2-*syn*) and 2’,3’,4’,6’-tetra-*O*-acetyl-α-ᴅ-galactopyranosyl-(1’→1)-2,3,4,5,6-penta-*O*-acetyl-7,8,9-trideoxy-ʟ-*glycero*-ʟ-*gulo*-8,9-nonenitol (2-*anti*):** Indium (263 mg, 2.29 mmol, 4 equiv) and allyl bromide (420 mg, 3.47 mmol, 6 equiv) were added to 20 mL of an ethanol/water solution (4:1, v/v) and heated to 65 °C under vigorous stirring. Subsequently, melibiose (**1**, 208 mg, 0.577 mmol, 1 equiv) was added and the reaction mixture was stirred at 65 °C for 4 h, until no further conversion of the starting material has been observed, indicated by TLC analysis (1-butanol/acetone/water 5:4:1, v/v/v). The reaction mixture was filtered over celite and the filtrate concentrated under reduced pressure, leaving a colorless oil. The crude material was dissolved in pyridine (10 mL) and treated with acetic anhydride (10 mL). A catalytic amount of DMAP was added at 0 °C and the reaction mixture was stirred for 3 h while the solution was allowed to come to room temperature (rt), until a single spot was detected by TLC analysis (heptane/ethyl acetate 1:2, v/v). The reaction mixture was diluted with toluene and the solvents evaporated under reduced pressure. The residue was separated in water and ethyl acetate, the aqueous phase was washed with ethyl acetate (3 × 5 mL), the combined organic layers were washed consecutively with water and brine, and dried over MgSO_4_. The solvent was evaporated under reduced pressure leading to a colorless highly viscous oil (405 mg, 0.531 mmol, 92%, **2-*syn***/**2-*anti*** 78:22). Separation of the resulting epimeric mixture was achieved via silica gel column chromatography (heptane/ethyl acetate 1:1, v/v).

Analytical data for (**2-*syn***): *R*_f_ = 0.6 (heptane/ethyl acetate 1:2, v/v); [α]_D_^20^ +83.3 (*c* 1.0, dichloromethane); mp 120–124 °C (methanol used for crystallization); ^1^H NMR (600.25 MHz, CDCl_3_, 25 °C) δ 5.653 (dddd, ^3^*J*_8,9a_ = 17.0 Hz, ^3^*J*_8,9b_ = 10.2 Hz, ^3^*J*_8,7b_ = 7.3 Hz, ^3^*J*_8,7a_ = 6.9 Hz, 1H, H-8), 5.449 (dd, ^3^*J*_4’,3’_ = 3.4 Hz, ^3^*J*_4’,5’_ = 1.2 Hz, 1H, H-4’), 5.416 (dd, ^3^*J*_3,2_ = 7.5 Hz, ^3^*J*_3,4_ = 3.3 Hz, 1H, H-3), 5.308 (dd, ^3^*J*_4,5_ = 7.8 Hz, ^3^*J*_4,3_ = 3.3 Hz, 1H, H-4), 5.304 (ddd, ^3^*J*_3’,2’_ = 11.0 Hz, ^3^*J*_3’,4’_ = 3.4 Hz, ^4^*J*_3’,5’_ = 0.3 Hz, 1H, H-3’), 5.193 (ddd, ^3^*J*_6,7b_ = 8.4 Hz, ^3^*J*_6,7a_ = 4.8 Hz, ^3^*J*_6,5_ = 3.4 Hz, 1H, H-6), 5.116 (dd, ^3^*J*_2’,3’_ = 11.0 Hz, ^3^*J*_2’,1’_ = 3.7 Hz, 1H, H-2’), 5.106 (dd, ^3^*J*_5,4_ = 7.8 Hz, ^3^*J*_5,6_ = 3.4 Hz, 1H, H-5), 5.089 (d, ^3^*J*_1’,2’_ = 3.7 Hz, 1H, H-1’), 5.066 (dddd, ^3^*J*_9a,8_ = 17.0 Hz, ^2^*J*_9a,9b_ = 1.8 Hz, ^4^*J*_9a,7a_ = 1.5 Hz, ^4^*J*_9a,7b_ = 1.5 Hz, 1H, H-9a), 5.063 (dddd, ^3^*J*_9b,8_ = 10.2 Hz, ^2^*J*_9b,9a_ = 1.8 Hz, ^4^*J*_9b,7a_ = 1.0 Hz, ^4^*J*_9b,7b_ = 1.0 Hz, 1H, H-9b), 4.962 (ddd, ^3^*J*_2,3_ = 7.5 Hz, ^3^*J*_2,1a_ = 5.6 Hz, ^3^*J*_2,1b_ = 3.8 Hz, 1H, H-2), 4.174 (dddd, ^3^*J*_5’,6’b_ = 7.2 Hz, ^3^*J*_5’,6’a_ = 6.1 Hz, ^3^*J*_5’,4’_ = 1.2 Hz, ^4^*J*_5’,3’_ = 0.3 Hz, 1H, H-5’), 4.079 (dd, ^2^*J*_6’a, 6’b_ = 11.2 Hz, ^3^*J*_6’a,5’_ = 7.2 Hz, 1H, H-6’a), 4.051 (dd, ^2^*J*_6’b, 6’a_ = 11.2 Hz, ^3^*J*_6’b,5’_ = 6.1 Hz, 1H, H-6’b), 3.679 (dd, ^2^*J*_1a,1b_ = 11.5 Hz, ^3^*J*_1a,2_ = 5.6 Hz, 1H, H-1a), 3.605 (dd, ^2^*J*_1b,1a_ = 11.5 Hz, ^3^*J*_1b,2_ = 3.8 Hz, 1H, H-1b), 2.265 (ddddd, ^2^*J*_7a,7b_ = 15.4 Hz, ^3^*J*_7a,8_ = 6.9 Hz, ^3^*J*_7a,6_ = 4.8 Hz, ^4^*J*_7a,9a_ = 1.5 Hz, ^4^*J*_7a,9b_ = 1.0 Hz, 1H, H-7a), 2.258 (ddddd, ^2^*J*_7b,7a_ = 15.4 Hz, ^3^*J*_7b,6_ = 8.4 Hz, ^3^*J*_7b,8_ = 7.3 Hz, ^4^*J*_7b,9a_ = 1.5 Hz, ^4^*J*_7b,9b_ = 1.0 Hz, 1H, H-7b), 2.125, 2.116, 2.093, 2.085, 2.085, 2.038, 2.034, 2.011, 1.955 (9s, 27H, 9 × OAc) ppm; ^13^C NMR (150.93 MHz, CDCl_3_, 25 °C) δ 170.57, 170.41, 170.15, 170.14, 169.81, 169.81, 169.78, 169.77, 169.49, (9 × O(*C*=O)CH_3_), 132.18 (C-8), 118.81 (C-9), 95,83 (C-1’), 70.35 (C-5), 70.03 (C-6), 68.87 (C-3), 68.74 (C-2), 68.37 (C-4), 67.67 (C-2’), 67.94 (C-4’), 67.28 (C-3’), 66.60 (C-5’), 65.44 (C-1), 61.59 (C-6’), 35.34 (C-7), 20.69, 20.66, 20.63, 20.63, 20.62, 20.61, 20.61, 20.56, 20.50 (9 × O(C=O)*C*H_3_) ppm; HRMS (ESI^+^) *m*/*z*: [M + Na]^+^ calcd for C_33_H_46_O_20_Na^+^, 785.2475; found, 785.2473. CCDC 1922520 contains the supplementary crystallographic data for compound **2-*syn***. These data can be obtained from The Cambridge Crystallographic Data Centre, http://www.ccdc.cam.ac.uk/data_request/cif.

Analytical data for (**2-*anti***): *R*_f_ = 0.7 (heptane/ethyl acetate 1:2, v/v); [α]_D_^20^ +64.1 (*c* 1.0, dichloromethane); ^1^H NMR (600.25 MHz, CDCl_3_, 25 °C) δ 5.729 (dddd, ^3^*J*_8,9a_ = 17.0 Hz, ^3^*J*_8,9b_ = 10.2 Hz, ^3^*J*_8,7a_ = 7.2 Hz, ^3^*J*_8,7b_ = 6.6 Hz, 1H, H-8), 5.468 (dd, ^3^*J*_4’,3’_ = 3.5 Hz, ^3^*J*_4’,5’_ = 1.3 Hz, 1H, H-4’), 5.396 (dd, ^3^*J*_4,5_ = 5.7 Hz, ^3^*J*_4,3_ = 5.1 Hz, 1H, H-4), 5.369 (dd, ^3^*J*_3,2_ = 6.3 Hz, ^3^*J*_3,4_ = 5.1 Hz, 1H, H-3), 5.308 (dd, ^3^*J*_3’,2’_ = 10.0 Hz, ^3^*J*_3’,4’_ = 3.5 Hz, 1H, H-3’), 5.301 (dd, ^3^*J*_5,4_ = 5.7 Hz, ^3^*J*_5,6_ = 5.5 Hz, 1H, H-5), 5.143 (dddd, ^3^*J*_9a,8_ = 17.0 Hz, ^2^*J*_9a,9b_ = 1.8 Hz, ^4^*J*_9a,7a_ = 1.4 Hz, ^4^*J*_9a,7b_ = 1.4 Hz, 1H, H-9a), 5.110 (d, ^3^*J*_1’,2’_ = 3.7 Hz, 1H, H-1’), 5.097 (dd, ^3^*J*_2’,3’_ = 10.0 Hz, ^3^*J*_2’,1’_ = 3.7 Hz, 1H, H-2’), 5.088 (dddd, ^3^*J*_9b,8_ = 10.2 Hz, ^2^*J*_9b,9a_ = 1.8 Hz, ^4^*J*_9b,7a_ = 1.1 Hz, ^4^*J*_9b,7b_ = 1.1 Hz, 1H, H-9b), 4.988 (ddd, ^3^*J*_2,3_ = 6.3 Hz, ^3^*J*_2,1a_ = 6.1 Hz, ^3^*J*_2,1b_ = 4.3 Hz, 1H, H-2), 4.936 (ddd, ^3^*J*_6,7b_ = 6.8 Hz, ^3^*J*_6,7a_ = 6.0 Hz, ^3^*J*_6,5_ = 5.5 Hz, 1H, H-6), 4.202 (ddd, ^3^*J*_5’,6’b_ = 6.7 Hz, ^3^*J*_5’,6’a_ = 6.5 Hz, ^3^*J*_5’,4’_ = 1.3 Hz, 1H, H-5’), 4.091 (dd, ^2^*J*_6’a, 6’b_ = 11.3 Hz, ^3^*J*_6’a,5’_ = 6.5 Hz, 1H, H-6’a), 4.063 (dd, ^2^*J*_6’b, 6’a_ = 11.3 Hz, ^3^*J*_6’b,5’_ = 6.7 Hz, 1H, H-6’b), 3.716 (dd, ^2^*J*_1a,1b_ = 11.3 Hz, ^3^*J*_1a,2_ = 6.1 Hz, 1H, H-1a), 3.615 (dd, ^2^*J*_1b,1a_ = 11.3 Hz, ^3^*J*_1b,2_ = 4.3 Hz, 1H, H-1b), 2.360 (ddddd, ^2^*J*_7a,7b_ = 15.0 Hz, ^3^*J*_7a,8_ = 6.6 Hz, ^3^*J*_7a,6_ = 6.0 Hz, ^4^*J*_7a,9a_ = 1.4 Hz, ^4^*J*_7a,9b_ = 1.1 Hz, 1H, H-7a), 2.355 (ddddd, ^2^*J*_7b,7a_ = 15.0 Hz, ^3^*J*_7b,8_ = 7.2 Hz, ^3^*J*_7b,6_ = 6.8 Hz, ^4^*J*_7b,9a_ = 1.4 Hz, ^4^*J*_7b,9b_ = 1.1 Hz, 1H, H-7b), 2.133, 2.122, 2.111, 2.102, 2.038, 2.026, 2.015, 1.962, 1.962 (9s, 27H, OAc) ppm; ^13^C NMR (150.93 MHz, CDCl_3_, 25 °C) δ 170.57, 170.36, 170.12, 170.11, 170.01, 169.98, 169.82, 169.69, 169.56 (9 × O(*C*=O)CH_3_), 132.67 (C-8), 118.54 (C-9), 96,09 (C-1’), 70.97 (C-5), 70.17 (C-6), 69.31 (C-3), 69.01 (C-2), 68.54 (C-4), 67.93 (C-2’), 67.93 (C-4’), 67.35 (C-3’), 66.64 (C-5’), 65.63 (C-1), 61.57 (C-6’), 34.09 (C-7), 20.75, 20.70, 20.70, 20.66, 20.65, 20.63, 20.59, 20.58, 20.53 (9 × O(C=O)*C*H_3_) ppm; HRMS (ESI^+^) *m*/*z*: [M + Na]^+^ calcd for C_33_H_46_O_20_Na^+^, 785.2475; found, 785.2479.

**2’,3’,4’,6’-Tetra-*O*-acetyl-α-ᴅ-galactopyranosyl-(1’→8)-1,3,4,6,7-penta-*O*-acetyl-2-deoxy-α-ᴅ-*glycero*-ᴅ-*ido*-octopyranose (5-*syn*-β):** Compound (**2-*syn***) (115 mg, 0.151 mmol) was dissolved in MeOH (15 mL) and treated with NaOMe (pH 9) until a pH around 9 was reached. The solution was stirred at rt for 2 h, until reaction monitoring via TLC analysis (1-butanol/acetone/H_2_O 5:4:1, v/v/v) showed complete conversion of the starting material. The reaction mixture was diluted with dichloromethane (dichloromethane/MeOH, 1:1, v/v) and subsequently cooled to −78 °C. Ozone was bubbled through the reaction with a gas inlet tube until a clear blue color was observed (2 min). Subsequently, the O_3_ generation was stopped allowing the gas flow to purge the reaction mixture for additional 15 min. PPh_3_ (80 mg, 0.305 mmol, 2 equiv) was added and stirring continued over night at rt. The solvents were evaporated under reduced pressure and the residue was separated between dichloromethane and water. The aqueous phase was washed with dichloromethane (3 × 10 mL, to separate PPh_3_). The water was removed under reduced pressure leaving a light yellow oil. The oil was diluted with pyridine (10 mL), and treated with acetic anhydride (10 mL) at 0 °C. A catalytic amount of DMAP was added and the reaction mixture was stirred for 3 h while the solution was allowed to come to room temperature until complete conversion could be observed on TLC analysis (heptane/ethyl acetate 1:2, v/v). The reaction mixture was diluted with toluene and the solvent removed under reduced pressure. The residue was separated between water and ethyl acetate and the aqueous phase was extracted with ethyl acetate (3 × 5 mL), the combined organic layers were washed subsequently with water, brine and dried over MgSO_4_. The solvent was removed under reduced pressure leaving a colorless highly viscous liquid. Separation of the resulting mixture of ring size and anomeric configuration was achieved via silica gel column chromatography (heptane/ethyl acetate 1:1, v/v) to give compound **5-*syn*-β** (48 mg, 0.063 mmol, 42%) as colorless oil. *R*_f_ = 0.5 (heptane/ethyl acetate 1:2, v/v); [α]_D_^20^ +88.2 (*c* 0.5, dichloromethane); ^1^H NMR (600.25 MHz, CDCl_3_, 25 °C) δ 5.791 (dd, ^3^*J*_1,2a_ = 10.4 Hz, ^3^*J*_1,2b_ = 2.2 Hz, 1H, H-1), 5.453 (dd, ^3^*J*_4’,3’_ = 3.4 Hz, ^3^*J*_4’,5’_ = 1.3 Hz, 1H, H-4’), 5.441 (dd, ^3^*J*_6,5_ = 6.7 Hz, ^3^*J*_6,7_ = 4.0 Hz, 1H, H-6), 5.280 (dd, ^3^*J*_3’,2’_ = 11.0 Hz, ^3^*J*_3’,4’_ = 3.4 Hz, 1H, H-3’), 5.129 (d, ^3^*J*_1’,2’_ = 3.7 Hz, 1H, H-1’), 5.103 (ddd, ^3^*J*_3,2a_ = 3.4 Hz, ^3^*J*_3,4_ = 3.1 Hz, ^3^*J*_3,2b_ = 2.8 Hz, 1H, H-3), 5.095 (dd, ^3^*J*_2’,3’_ = 11.0 Hz, ^3^*J*_2’,1’_ = 3.7 Hz, 1H, H-2’), 5.000 (ddd, ^3^*J*_7,8a_ = 7.3 Hz, ^3^*J*_7,6_ = 4.0 Hz, ^3^*J*_7,8b_ = 3.2 Hz, 1H, H-7), 4.790 (dd, ^3^*J*_4,3_ = 3.1 Hz, ^3^*J*_4,5_ = 1.5 Hz, 1H, H-4), 4.186 (ddd, ^3^*J*_5’,6’b_ = 7.1 Hz, ^3^*J*_5’,6’a_ = 6.4 Hz, ^3^*J*_5’,4’_ = 1.3 Hz, 1H, H-5’), 4.119 (dd, ^3^*J*_5,6_ = 6.7 Hz, ^3^*J*_5,4_ = 1.5 Hz, 1H, H-5), 4.084 (dd, ^2^*J*_6’a,6’b_ = 12.0 Hz, ^3^*J*_6’a,5’_ = 6.4 Hz, 1H, H-6’a), 4.083 (dd, ^2^*J*_6’b,6’a_ = 12.0 Hz, ^3^*J*_6’b,5’_ = 7.1 Hz, 1H, H-6’b), 3.813 (dd, ^2^*J*_8a, 8b_ = 11.7 Hz, ^3^*J*_8a,7_ = 7.3 Hz, 1H, H-8a), 3.695 (dd, ^2^*J*_8b,8a_ = 11.7 Hz, ^3^*J*_8b,7_ = 3.2 Hz, 1H, H-8b), 2.174, 2.134, 2.128, 2.103, 2.087, 2.086, 2.045, 2.043 (8s, 24H, OAc), 2.026 (ddd, ^2^*J*_2a,2b_ = 14.3 Hz, ^3^*J*_2a,1_ = 10.4 Hz, ^3^*J*_2a,3_ = 3.4 Hz, 1H, H-2a), 1.978 (1s, 3H, OAc), 1.948 (ddd, ^2^*J*_2b,2a_ = 14.3 Hz, ^3^*J*_2b,3_ = 2.8 Hz, ^3^*J*_2b,1_ = 2.2 Hz, 1H, H-2b) ppm; ^13^C NMR (150.93 MHz, CDCl_3_, 25 °C) δ 170.59, 170.34, 170.17, 169.93, 169.92, 169.82, 169.79, 169.08, 168.92 (O(*C*=O)CH_3_), 96.33 (C-1’), 90.47 (C-1), 72.31 (C-5), 70.76 (C-7), 70.36 (C-6), 67.93 (C-2’), 67.79 (C-4’), 67.69 (C-3), 67.37 (C-3’), 66.45 (C-5’), 65.77 (C-4), 65.74 (C-8), 61.35 (C-6’), 29.95 (C-2), 20.97, 20.92, 20.80, 20.74, 20.72, 20.70, 20.66, 20.66, 20.62 (9 × O(C=O)*C*H_3_) ppm; HRMS (ESI^+^) *m*/*z*: [M + NH_4_]^+^ calcd for C_32_H_48_NO_21_^+^, 782.2713; found, 782.2719.

**2’,3’,4’,6’-Tetra-*O*-acetyl-α-ᴅ-galactopyranosyl-(1’→8)-1,3,4,6,7-penta-*O*-acetyl-2-deoxy-α-ᴅ-*glycero*-ᴅ-*gulo*-octopyranose (5-*anti*-β):** Compound **2-*anti*** (154 mg, 0.202 mmol) was reacted accordingly as described for the conversion of **2-*syn***, leading to a colorless highly viscous oil. Separation of the resulting mixture of ring size and anomeric configuration was achieved via silica gel chromatography (heptane/ethyl acetate 1:1, v/v) to give compound **5-*anti*-β** (62 mg, 0.081 mmol, 40%) as colorless oil. *R*_f_ = 0.5 (heptane/ethyl acetate 1:2, v/v); [α]_D_^20^ +36.7 (*c* 1.0, dichloromethane); ^1^H NMR (600.25 MHz, CDCl_3_, 25 °C) δ 5.674 (dd, ^3^*J*_1,2a_ = 10.1 Hz, ^3^*J*_1,2b_ = 2.2 Hz, 1H, H-1), 5.539 (dd, ^3^*J*_4’,3’_ = 3.5 Hz, ^3^*J*_4’,5’_ = 1.0 Hz, 1H, H-4’), 5.524 (dd, ^3^*J*_4,3_ = 3.0 Hz, ^3^*J*_4,5_ = 1.0 Hz, 1H, H-4), 5.511 (dd, ^3^*J*_3’,2’_ = 10.8 Hz, ^3^*J*_3’,4’_ = 3.5 Hz, 1H, H-3’), 5.494 (dd, ^3^*J*_6,5_ = 8.5 Hz, ^3^*J*_6,7_ = 1.9 Hz, 1H, H-6), 5.353 (ddd, ^3^*J*_3,2a_ = 12.6 Hz, ^3^*J*_3,2b_ = 5.0 Hz, ^3^*J*_3,4_ = 3.0 Hz, 1H, H-3), 5.156 (d, ^3^*J*_1’,2’_ = 4.0 Hz, 1H, H-1’), 5.095 (dd, ^3^*J*_2’,3’_ = 10.8 Hz, ^3^*J*_2’,1’_ = 4.0 Hz, 1H, H-2’), 4.988 (ddd, ^3^*J*_7,8b_ = 9.7 Hz, ^3^*J*_7,8a_ = 5.3 Hz, ^3^*J*_7,6_ = 1.9 Hz, 1H, H-7), 4.312 (ddd, ^3^*J*_5’,6’a_ = 6.8 Hz, ^3^*J*_5’,6’b_ = 6.0 Hz, ^3^*J*_5’,4’_ = 1.0 Hz, 1H, H-5’), 4.232 (dd, ^3^*J*_5,6_ = 8.5 Hz, ^3^*J*_5,4_ = 1.0 Hz, 1H, H-5), 4.124 (dd, ^2^*J*_6’a,6’b_ = 11.7 Hz, ^3^*J*_6’a,5’_ = 6.8 Hz, 1H, H-6’a), 4.114 (dd, ^2^*J*_6’b,6’a_ = 11.7 Hz, ^3^*J*_6’b,5’_ = 6.0 Hz, 1H, H-6’b), 3.819 (dd, ^2^*J*_8a, 8b_ = 8.7 Hz, ^3^*J*_8a,7_ = 5.3 Hz, 1H, H-8a), 3.656 (dd, ^3^*J*_8b,7_ = 9.7 Hz, ^2^*J*_8b,8a_ = 8.7 Hz, 1H, H-8b), 2.256, 2.185, 2.153 (3s, 9H, OAc), 2.105 (ddd, ^2^*J*_2a,2b_ = 14.3 Hz, ^3^*J*_2a,3_ = 12.6 Hz, ^3^*J*_2a,1_ = 10.1 Hz, 1H, H-2a), 2.088, 2.064, 2.056 (3s, 9H, OAc), 1.980 (ddd, ^2^*J*_2b,2a_ = 14.3 Hz, ^3^*J*_2b,3_ = 5.0 Hz, ^3^*J*_2b,1_ = 2.2 Hz, 1H, H-2b), 1.975, 1.975, 1.959 (3s, 9H, OAc) ppm; ^13^C NMR (150.93 MHz, CDCl_3_, 25 °C) δ 171.07, 170.54, 170.44, 170.02, 169.96, 169.88, 169.56, 169.34, 168.96 (9 × O(*C*=O)CH_3_), 97.02 (C-1’), 91.46 (C-1), 73.48 (C-5), 71.33 (C-6), 68.99 (C-2’), 68.49 (C-4’), 68.43 (C-3), 68.11 (C-7), 67.22 (C-3’), 67.15 (C-5’), 65.88 (C-8), 64.90 (C-4), 61.68 (C-6’), 30.31 (C-2), 20.96, 20.88, 20.77, 20.73, 20.71, 20.69, 20.66, 20.64, 20.58 (9 × O(C=O)*C*H_3_) ppm; HRMS (ESI^+^) *m*/*z*: [M + NH_4_]^+^ calcd for C_32_H_48_NO_21_^+^, 782.2713; found, 782.2720.

## Supporting Information

Supporting information features copies of ^1^H NMR, ^13^C NMR spectra and mass analysis data of all compounds, as well as X-ray data of compound **2-*syn***.

File 1 General instructions, NMR spectra, mass analysis data and X-ray data.

File 2 Cif-report of compound **2-*syn****.*

File 3 Crystallographic information file of compound **2-*syn****.*
