# Supplementary material for: Indium-mediated C-allylation of melibiose
Source: Beilstein J Org Chem. 2019 Oct 16;15:2458–64. doi: 10.3762/bjoc.15.238 (PMC6808214; doi:10.3762/bjoc.15.238)
Supplement: File 1 — General instructions, NMR spectra, mass analysis data and X-ray data. [file Beilstein_J_Org_Chem-15-2458-s001.pdf]

BEILSTEIN JOURNAL OF ORGANIC CHEMISTRY

Supporting Information

for

Indium-mediated C-allylation of melibiose

Christian Denner, Manuel Gintner, Hanspeter Kählig and Walther Schmid

Beilstein J. Org. Chem. **2019,** *15,* 2458–2464. doi:10.3762/bjoc.15.238

General instructions, NMR spectra, mass analysis data and X-ray data

License and Terms: This is a supporting information file under the terms of the Creative Commons Attribution License (http://creativecommons.org/licenses/by/4.0). Please note that the reuse, redistribution and reproduction in particular requires that the authors and source are credited.
The license is subject to the *Beilstein Journal of Organic Chemistry* terms and conditions: (https://www.beilstein-journals.org/bjoc)

Table of Contents

General instructions………………………………………….. ………….. S2

^{1}H NMR and ^{13}C NMR spectra…………………………………………….. S3

HRMS spectra………………………………………………………………. S7

X-ray analysis………………………………………………………………. S11

General instructions: ^{1}H and ^{13}C NMR spectra were recorded on a Bruker AV III 600. Chemical shifts (δ) are reported in parts per million (ppm) and spectra were calibrated using solvent signal of $CDCl_3$. Signal multiplicity is indicated by one or more of the following: s (singlet); d (doublet); t (triplet); q (quartet). NMR analysis was assisted by the spin simulation software DAISY (part of Bruker Top Spin software). High resolution mass spectra (HRMS) were recorded on a Bruker maXis UHR-TOF spectrometer in ESI mode. Optical rotations were measured on a Schmidt-Haensch Digital Polarimeter Unipol L 2000. Ozonolysis was performed operating an Anseros COM-AD-04 ozone generator. Column chromatography was performed using Macherey-Nagel silica gel 60 (0.04–0.063 mm, 240–400 mesh). TLC monitoring was carried out on precoated Merck silica gel 60 F_{254} glass plates. Compounds were visualized by treatment with a Mo–$Ce(SO_4)_2$ complex solution (48 g $(NH_4)_6Mo_7O_{24}$ and 2 g $CeSO_4$ in 1 dm^3 10% H_2SO_4) followed by heating. Filtrations over celite were performed with Celite® S by Sigma-Aldrich®. Solvents were distilled, if necessary, prior to use. Reagents were purchased from commercial suppliers and were used without further purification.

2’,3’,4’,6’-Tetra-*O*-acetyl-α-D-galactopyranosyl-(1’→1)-2,3,4,5,6-penta-*O*-acetyl-7,8,9-trideoxy-D-*glycero*-L-*gulo*-8,9-nonenitol (**2-*syn***)

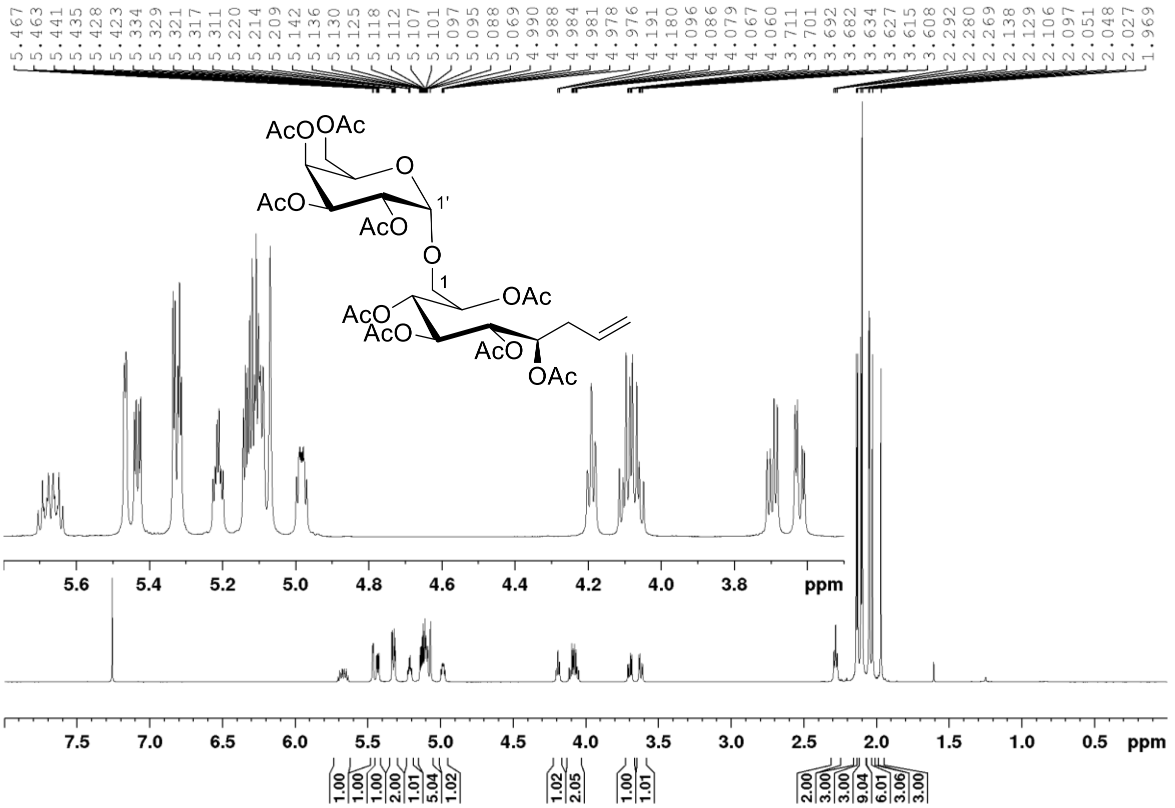

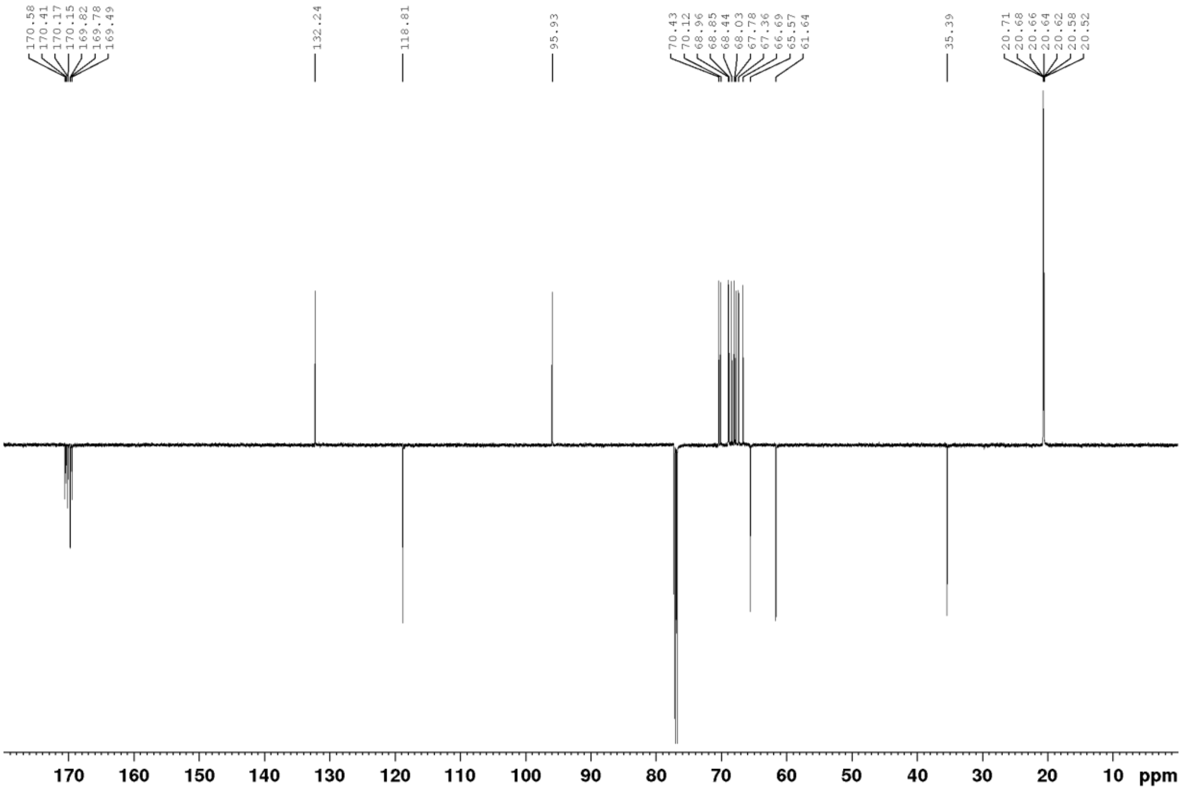

2’,3’,4’,6’-Tetra-*O*-acetyl-α-D-galactopyranosyl-(1’→1)-2,3,4,5,6-penta-*O*-acetyl-7,8,9-trideoxy-L-*glycero*-L-*gulo*-8,9-nonenitol (**2-*anti***)

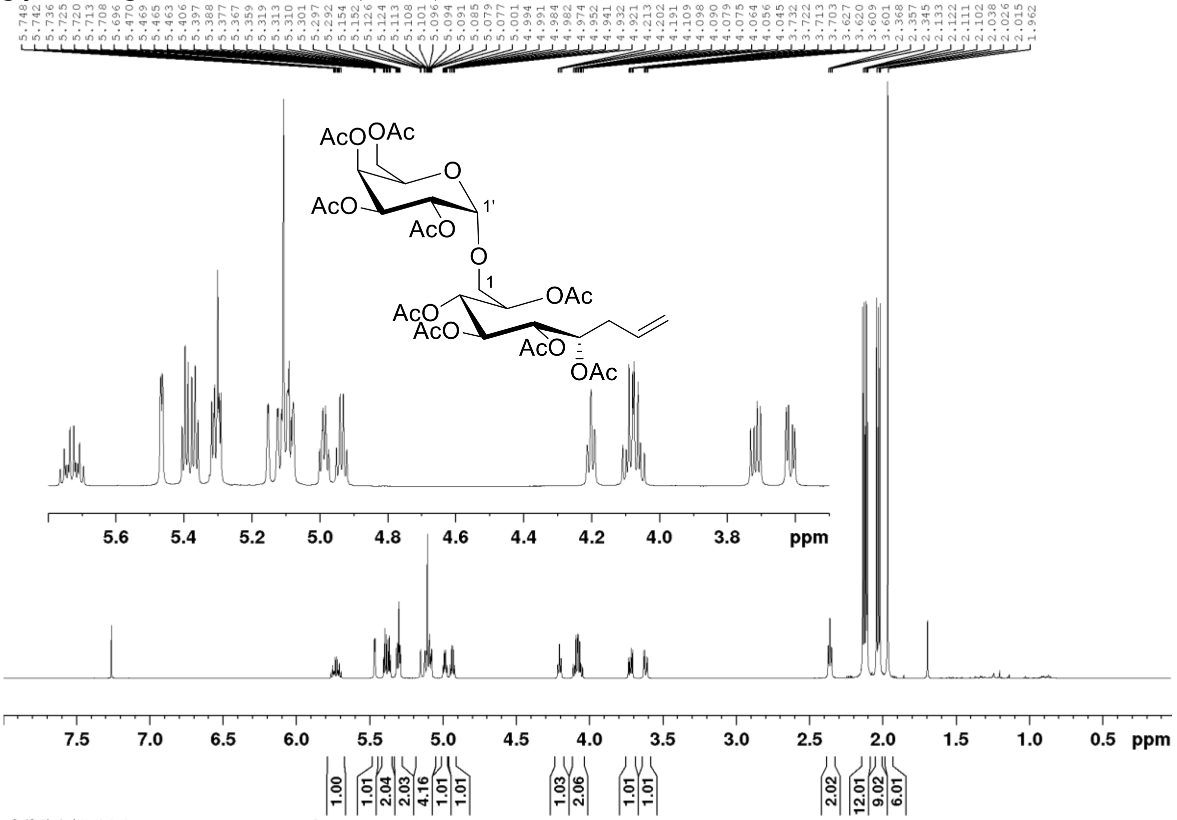

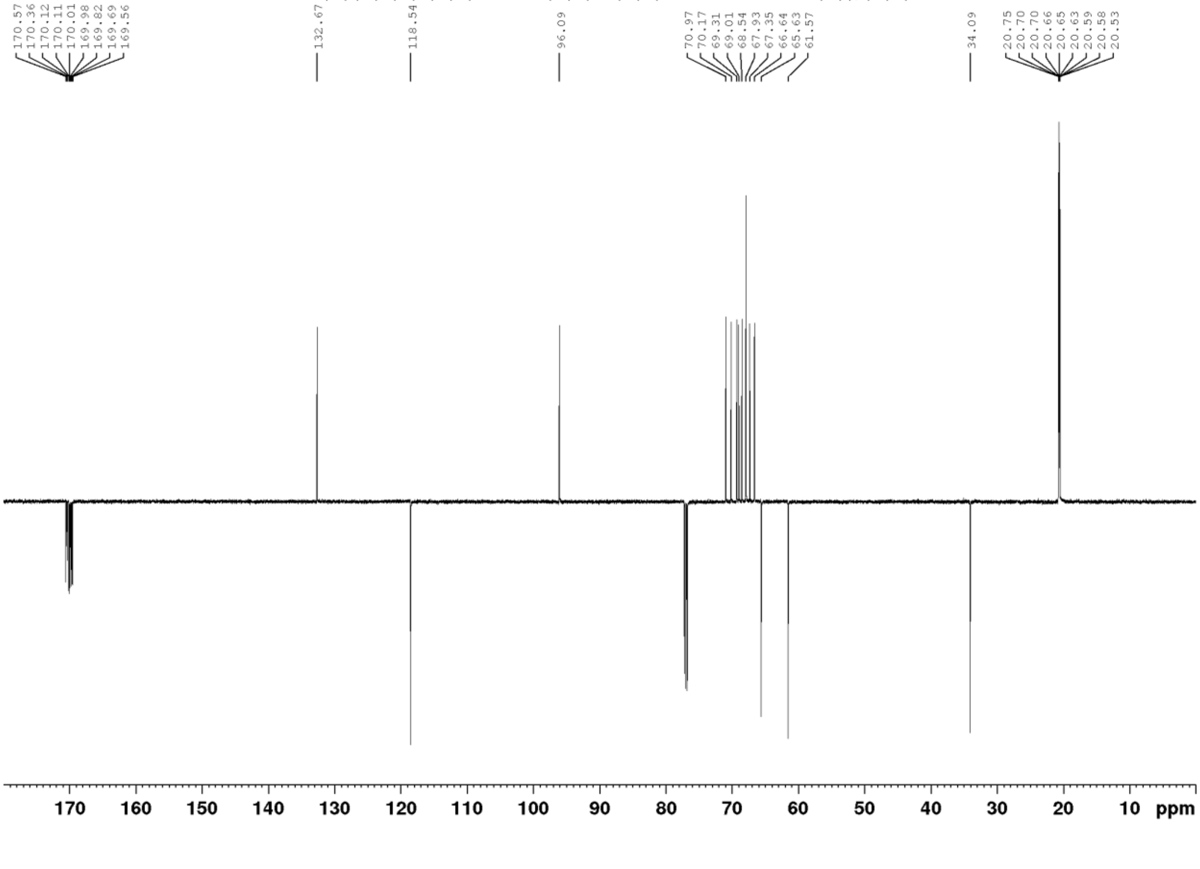

2',3',4',6'-Tetra-*O*-acetyl-α-D-galactopyranosyl-(1'→8)-1,3,4,6,7-penta-*O*-acetyl-2-deoxy-α-D-*glycero*-D-*ido*-octopyranose (**5-*syn*-β**)

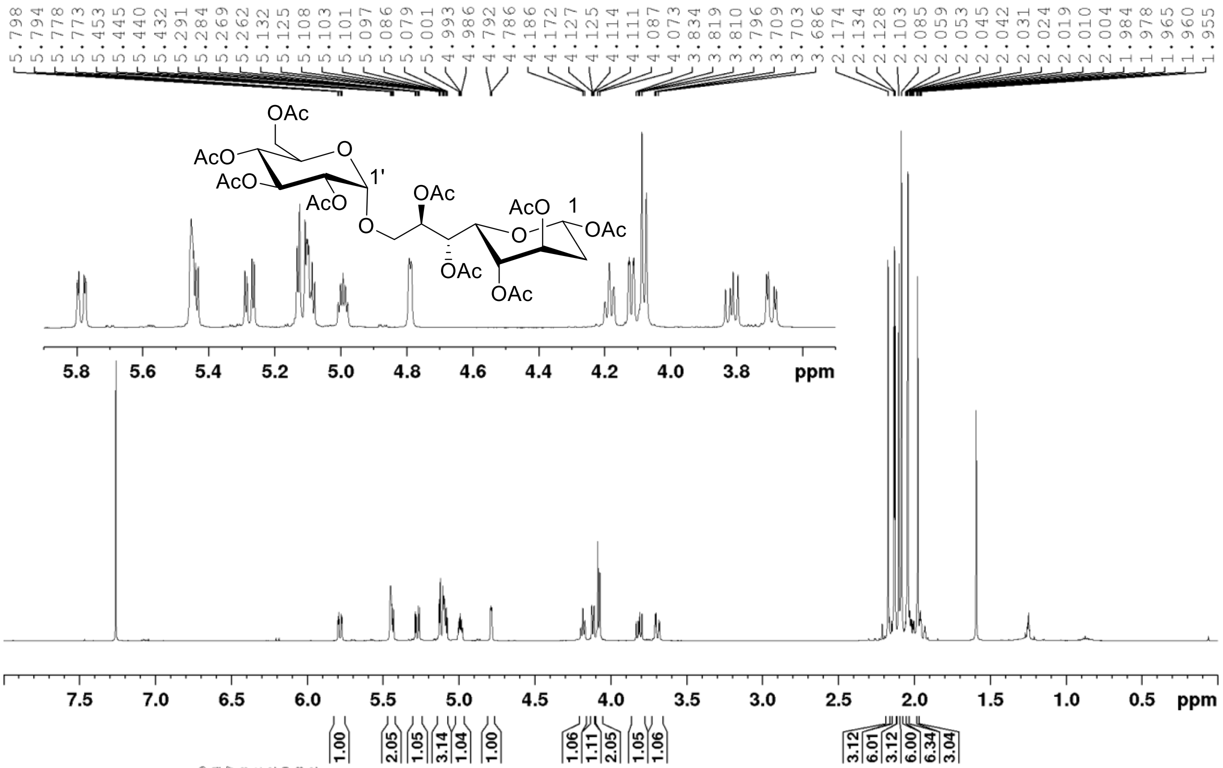

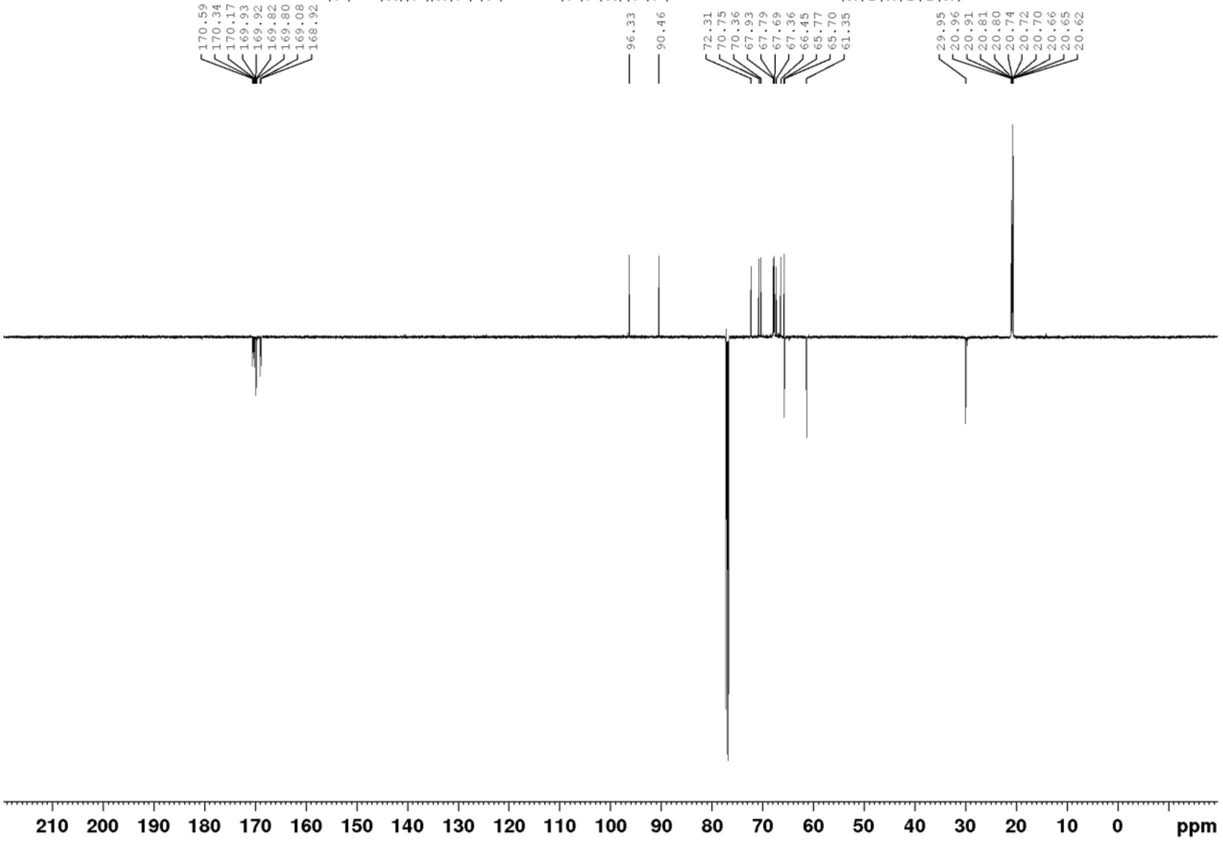

2’,3’,4’,6’-Tetra-*O*-acetyl-α-D-galactopyranosyl-(1’→8)-1,3,4,6,7-penta-*O*-acetyl-2-deoxy-α-D-*glycero*-D-*gulo*-octopyranose (**5-*anti*-β**)

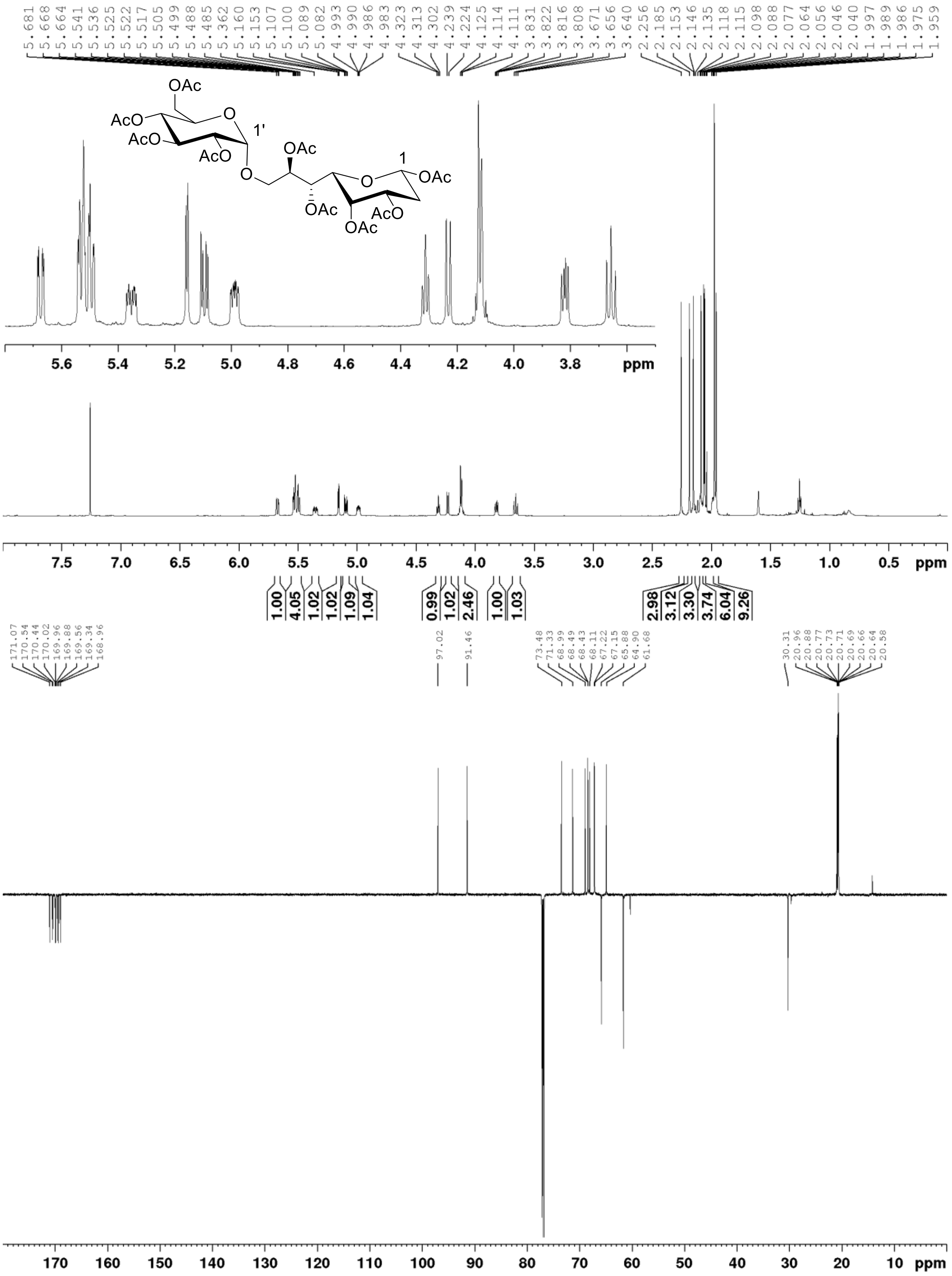

2’,3’,4’,6’-Tetra-*O*-acetyl-α-D-galactopyranosyl-(1’→1)-2,3,4,5,6-penta-*O*-acetyl-7,8,9-trideoxy-D-*glycero*-L-*gulo*-8,9-nonenitol (**2-*syn***)

HRMS (ESI+) m/z = 785.2473 [M+Na]+ calcd. for $C_{33}H_{46}O_{20}Na^{+}$: 785.2475.

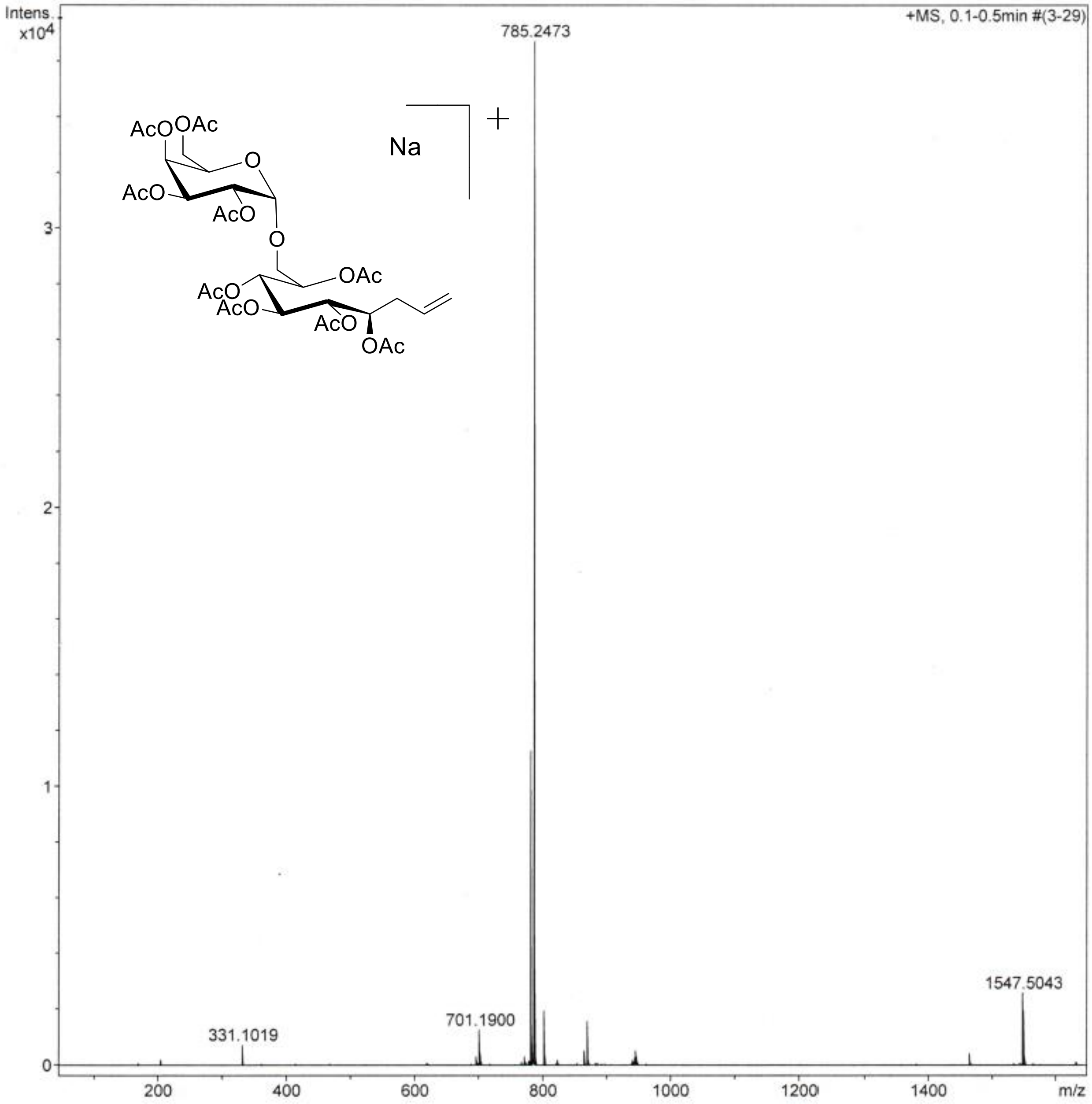

2',3',4',6'-Tetra-*O*-acetyl-α-D-galactopyranosyl-(1'→1)-2,3,4,5,6-penta-*O*-acetyl-7,8,9-trideoxy-L-*glycero*-L-*gulo*-8,9-nonenitol (**2-*anti***)

HRMS (ESI$^+$) m/z = 785.2479 [M+Na]$^+$ calcd. for $C_{33}H_{46}O_{20}Na^+$: 785.2475.

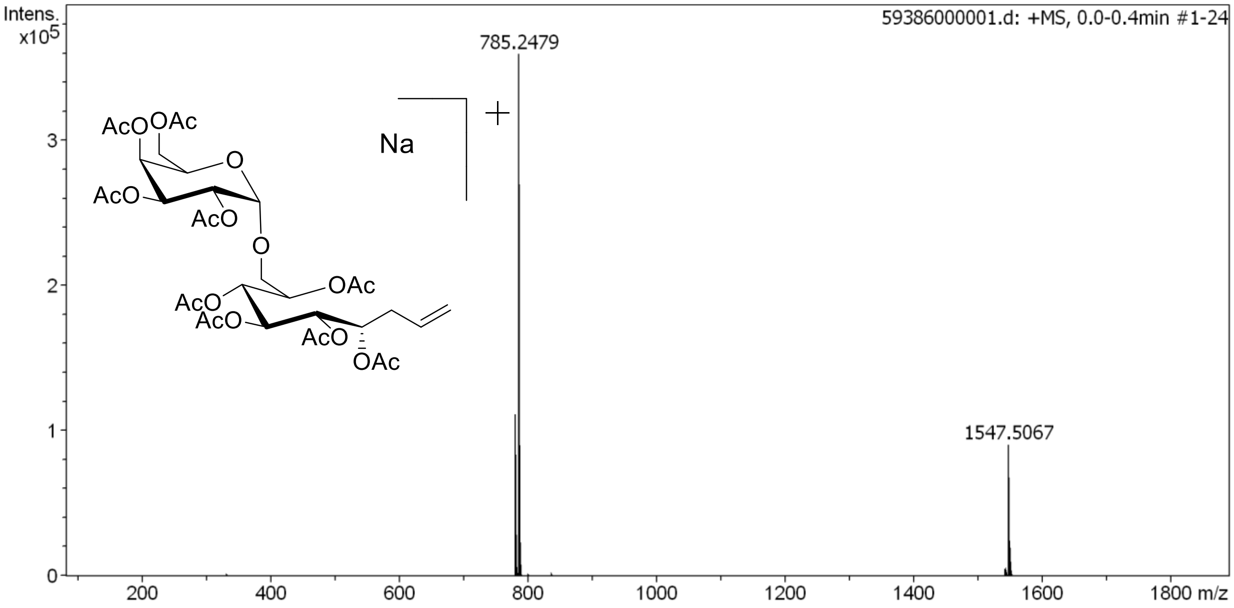

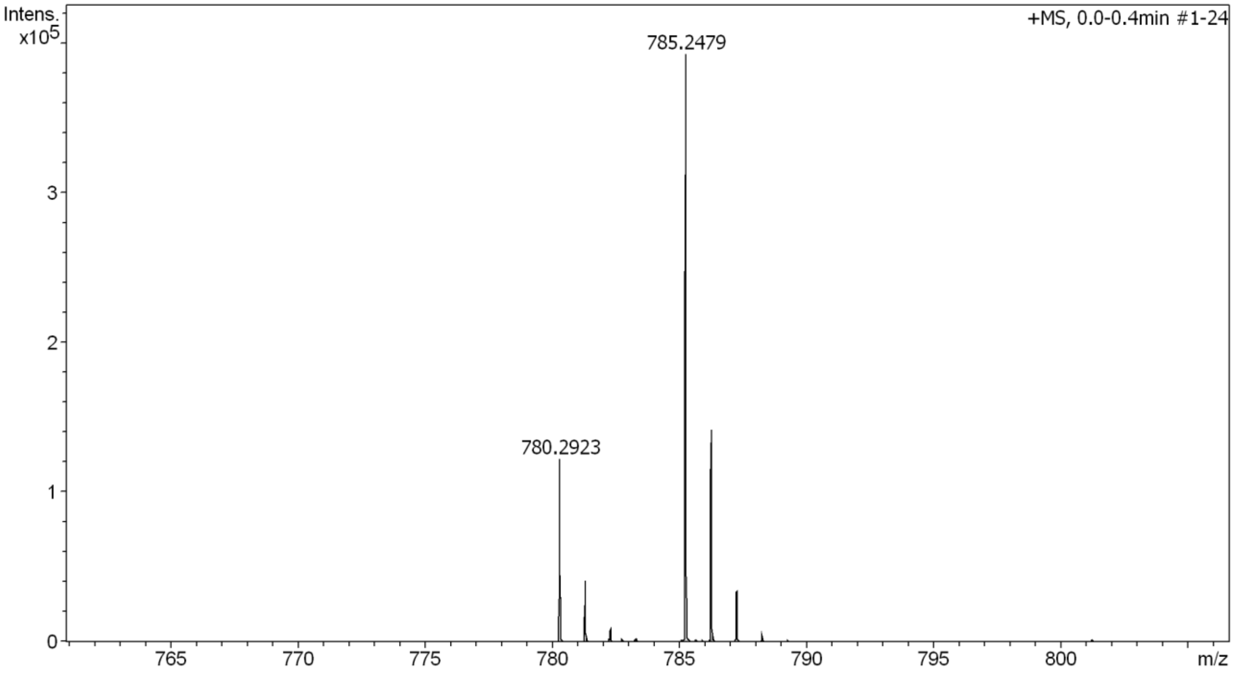

2’,3’,4’,6’-Tetra-*O*-acetyl-α-D-galactopyranosyl-(1’→8)-1,3,4,6,7-penta-*O*-acetyl-2-deoxy-α-D-*glycero*-D-*ido*-octopyranose (**5-*syn*-β**)

HRMS (ESI$^+$) m/z = 782.2719 [M+ NH$_4$]$^+$ calcd. for $C_{32}H_{48}NO_{21}^+$: 782.2713.

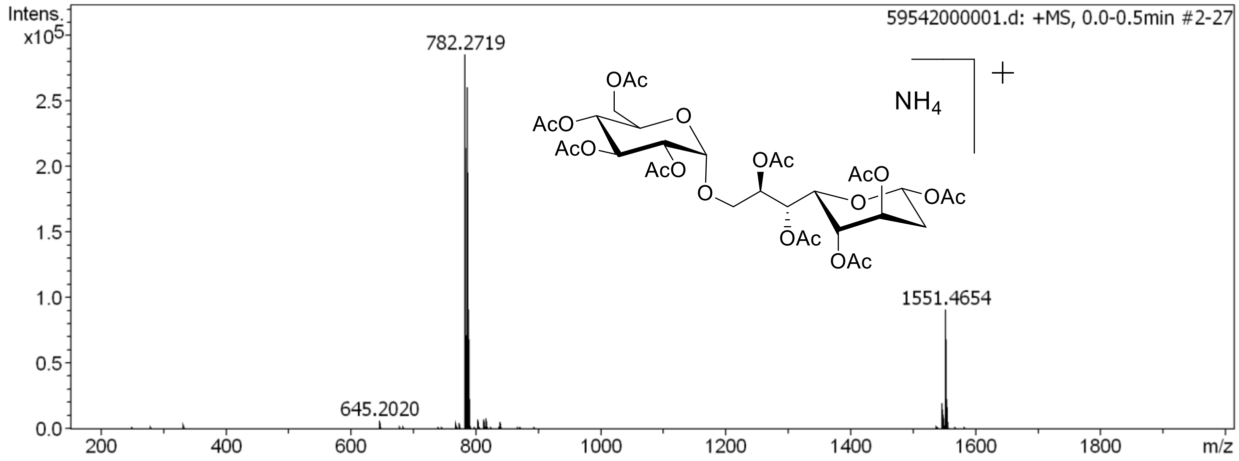

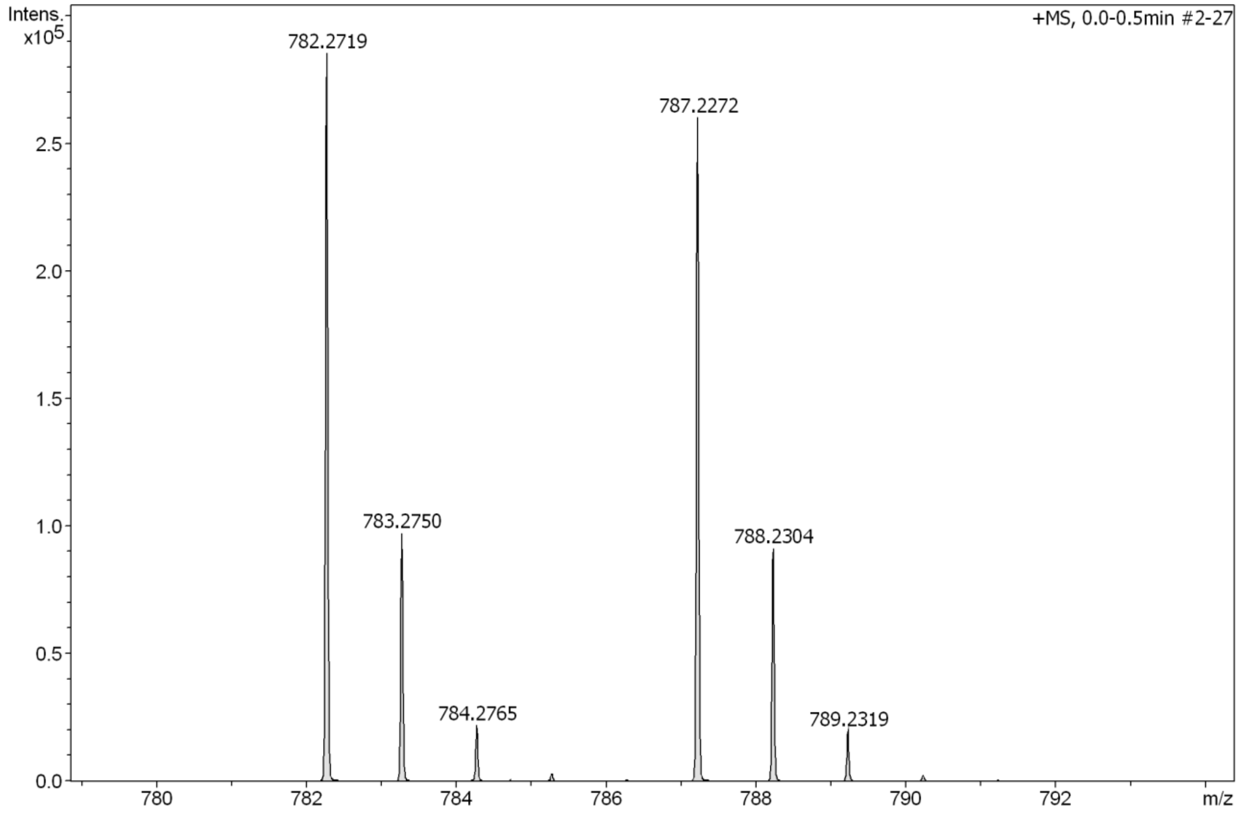

2’,3’,4’,6’-Tetra-*O*-acetyl-α-D-galactopyranosyl-(1’→8)-1,3,4,6,7-penta-*O*-acetyl-2-deoxy-α-D-*glycero*-D-*gulo*-octopyranose (**5-*anti*-β**)

HRMS (ESI^+) m/z = 782.2720 $[M+NH_4]^+$ calcd. for $C_{32}H_{48}NO_{21}^+$: 782.2713.

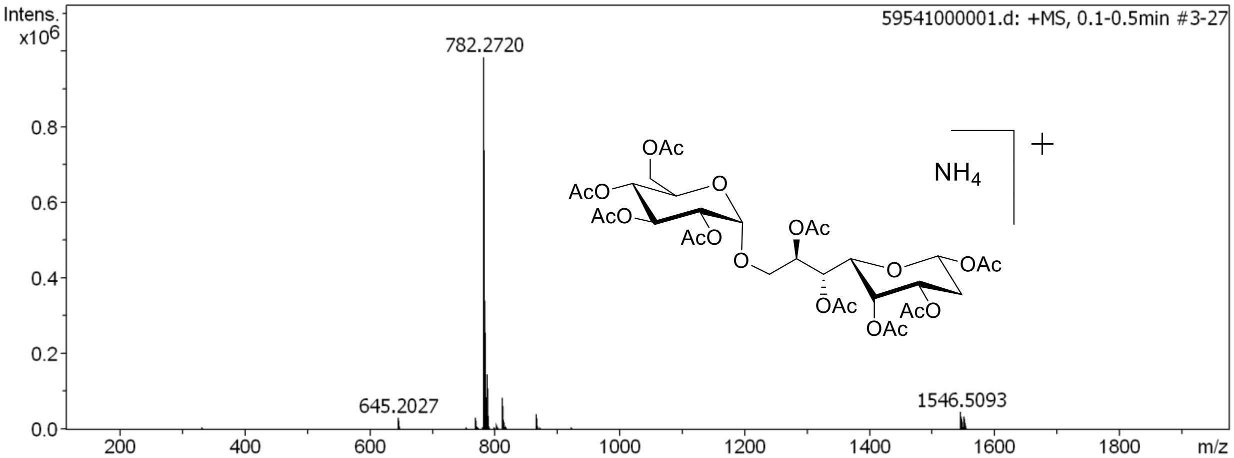

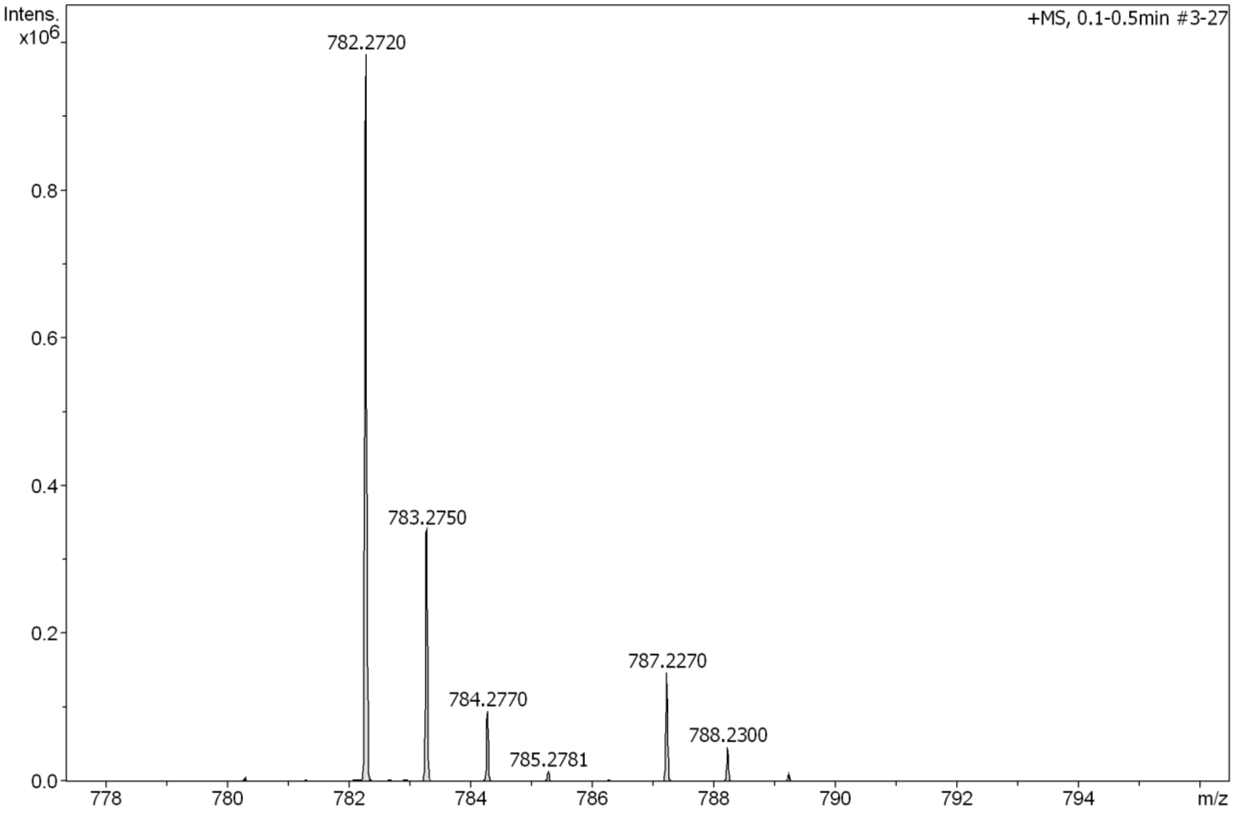

X-ray analysis

The X-ray intensity data were measured on Bruker D8 Venture diffractometer equipped with multilayer monochromator, Mo Kα INCOATEC micro focus sealed tube and Oxford cooling system. The structure was solved by *charge flipping* and refined by *full-matrix least-squares techniques*. Non-hydrogen atoms were refined with *anisotropic displacement parameters*. Hydrogen atoms were inserted at calculated positions and refined with riding model. The following software was used: *Bruker SAINT software package*[i] using a narrow-frame algorithm for frame integration, *SADABS*[ii] for absorption correction, *OLEX2*[iii] for structure solution, molecular diagrams and graphical user-interface, *Shelxle*[iv] for refinement and graphical user-interface, *SHELXL-2015*[v] for refinement, *Platon*[vi] for symmetry check. Experimental data and CCDC-Codes Experimental data and CCDC-Code (Available online: http://www.ccdc.cam.ac.uk/conts/retrieving.html) can be found in Table S1. Crystal data, data collection parameters, and structure refinement details are given in Tables S2 and S3. Crystal structure is visualized in Figure S1.

Table S1 Experimental parameter and CCDC-Code.

**Sample**	**Machine**	**Source**	**Temp.**	**Detector Distance**	**Time/ Frame**	**#Frames**	**Frame width**	**CCDC**
	Bruker		[K]	[mm]	[s]		[°]	
**2-syn**	D8	Mo	100	37	8	6216	0.500	1922520

2-syn

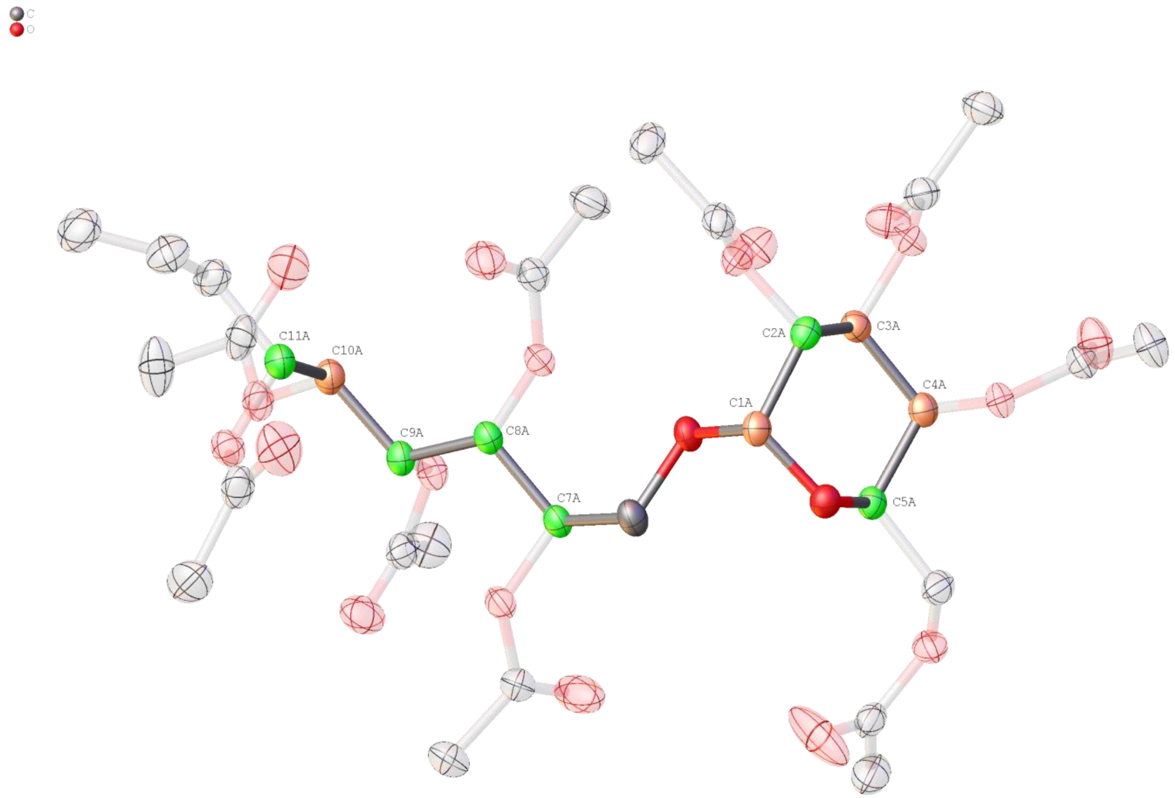

Figure S1 Crystal structure of [**2-syn**], drawn with 50% displacement ellipsoid. Backbone highlighted, side chains translucent. The green carbons are in R conformation, the orange ones are S. Chirality is proofed by reference molecule and AD. The bond precision for C–C single bonds is 0.0033 Å. Second independent molecule in the AU, Hydrogen Atoms and Disorder omitted for clarity. Main residue disorder is 9%.

Table S2 Sample and crystal data [**2-syn**].

**Chemical formula**	C33H46O20	**Crystal system**	monoclinic	
**Formula weight [g/mol]**	762.7	**Space group**	*P21*	
**Temperature [K]**	100	**Z**	4	
**Measurement method**	\f and \w scans	**Volume [Å^3]**	3800.8(3)	
**Radiation (Wavelength [Å])**	MoKα (λ = 0.71073)	**Unit cell dimensions [Å] and [°]**	14.1418(6)	90
**Crystal size / [mm^3]**	0.35 × 0.23 × 0.211		9.0704(3)	91.8306(15)
**Crystal habit**	clear colourless block		29.6461(12)	90
**Density (calculated) / [g/cm^3]**	1.333	**Absorption coefficient / [mm^{-1}]**	0.111	
**Abs. correction Tmin**	0.7191	**Abs. correction Tmax**	0.746	
**Abs. correction type**	multiscan	**F(000) [e$^-$]**	1616	

Table S3 Data collection and structure refinement[**2-syn**]..

**Index ranges**	-19 ≤ h ≤ 19, -12 ≤ k ≤ 12, -41 ≤ l ≤ 41	**Theta range for data collection [°]**	4.124 to 60.124	
**Reflections number**	489568	**Data / restraints / parameters**	22249/5/1051	
**Refinement method**	Least squares	**Final R indices**	all data	R1 = 0.0483, wR2 = 0.1031
**Function minimized**	$\Sigma\ w(F_o^2 - F_c^2)^2$		I>2σ(I)	R1 = 0.0384, wR2 = 0.0925
**Goodness-of-fit on F^2**	1.093	**Weighting scheme**	$w=1/[\sigma^2(F_o^2)+(0.0403P)^2+1.2026P]$	
**Largest diff. peak and hole [e Å^{-3}]**	0.47/-0.25		where $P=(F_o^2+2F_c^2)/3$	

[i] Bruker SAINT v8.38B Copyright © 2005-2019 Bruker AXS
[ii] Sheldrick, G. M. (1996). *SADABS*. University of Göttingen, Germany.
[iii] Dolomanov, O.V.; Bourhis, L.J.; Gildea, R.J; Howard, J.A.K.; Puschmann, H. OLEX2, (2009), *J. Appl. Cryst.* **2009,** *42,* 339-341
[iv] Huebschle, C. B.; Sheldrick, G. M.; Dittrich, B. ShelXle: a Qt graphical user interface for SHELXL, *J. Appl. Cryst.* **2011,** *44,* 1281-1284
[v] Sheldrick, G. M. (2015). *SHELXL* v 2016/4 University of Göttingen, Germany.
[vi] Spek, A. L. *Acta Cryst.* **2009,** *D65,* 148-155.
