# Supplementary material for: Indium-mediated C-allylation of melibiose
Source: Beilstein J Org Chem. 2019 Oct 16;15:2458–64. doi: 10.3762/bjoc.15.238 (PMC6808214; doi:10.3762/bjoc.15.238)
Supplement: File 2 — Cif-report of compound 2-syn. [file Beilstein_J_Org_Chem-15-2458-s002.pdf]

BEILSTEIN JOURNAL OF ORGANIC CHEMISTRY

Supporting Information

for

Indium-mediated C-allylation of melibiose

Christian Denner, Manuel Gintner, Hanspeter Kählig and Walther Schmid

Beilstein J. Org. Chem. **2019,** *15,* 2458–2464. doi:10.3762/bjoc.15.238

Cif-report of compound 2-*syn*

License and Terms: This is a supporting information file under the terms of the Creative Commons Attribution License (http://creativecommons.org/licenses/by/4.0). Please note that the reuse, redistribution and reproduction in particular requires that the authors and source are credited. The license is subject to the *Beilstein Journal of Organic Chemistry* terms and conditions: (https://www.beilstein-journals.org/bjoc)

No syntax errors found.
Please wait while processing ....

CIF dictionary
Interpreting this report

Datablock: chde_mehle_p21

Bond precision:	C-C = 0.0033 A		Wavelength=0.71073
Cell:	a=14.1418(6)	b=9.0704(3)	c=29.6461(12)
	alpha=90	beta=91.8306 (15)	gamma=90
Temperature:	100 K		

	Calculated	Reported
Volume	3800.8(3)	3800.8(3)
Space group	P 21	P 21
Hall group	P 2yb	P 2yb
Moiety formula	C33 H46 O20	1(C33 H46 O20)
Sum formula	C33 H46 O20	C33 H46 O20
Mr	762.70	762.70
Dx,g cm-3	1.333	1.333
Z	4	4
Mu (mm-1)	0.111	0.111
F000	1616.0	1616.0
F000'	1617.12	
h,k,lmax	19,12,41	19,12,41
Nref	22306[11803]	22249
Tmin,Tmax	0.970,0.977	0.719,0.746
Tmin'	0.962	

Correction method= # Reported T Limits: Tmin=0.719
Tmax=0.746 AbsCorr = MULTI-SCAN

Data completeness= 1.89/1.00 Theta(max)= 30.062

R(reflections)= 0.0384(19783) wR2(reflections)= 0.1031(22249)

S = 1.093 Npar= 1051

The following ALERTS were generated. Each ALERT has the format
test-name_ALERT_alert-type_alert-level.
Click on the hyperlinks for more details of the test.

Alert level C

PLAT220_ALERT_2_C Non-Solvent Resd 2 O Ueq(max)/Ueq(min) Range 3.1 Ratio

Alert level G

PLAT002_ALERT_2_G Number of Distance or Angle Restraints on AtSite 9 Note
PLAT012_ALERT_1_G No _shelx_res_checksum Found in CIF ...... Please Check
PLAT042_ALERT_1_G Calc. and Reported MoietyFormula Strings Differ Please Check
PLAT171_ALERT_4_G The CIF-Embedded .res File Contains EADP Records 1 Report
PLAT176_ALERT_4_G The CIF-Embedded .res File Contains SADI Records 4 Report
PLAT301_ALERT_3_G Main Residue Disorder ....(Resd 1) 9% Note
PLAT301_ALERT_3_G Main Residue Disorder ....(Resd 2) 8% Note
PLAT380_ALERT_4_G Incorrectly? Oriented X(sp2)-Methyl Moiety ..... C27A Check
PLAT398_ALERT_2_G Deviating C-O-C Angle From 120 for O2B 109.7 Degree
PLAT432_ALERT_2_G Short Inter X...Y Contact O10Y ..C29B 2.79 Ang.
x,y,z = 1_555 Check
PLAT720_ALERT_4_G Number of Unusual/Non-Standard Labels .... 4 Note
PLAT791_ALERT_4_G Model has Chirality at C1A (Chiral SPGR) S Verify
And 14 other PLAT791 Alerts

PLAT791_ALERT_4_G Model has Chirality at C1B (Chiral SPGR) S Verify
PLAT791_ALERT_4_G Model has Chirality at C2A (Chiral SPGR) R Verify
PLAT791_ALERT_4_G Model has Chirality at C2B (Chiral SPGR) R Verify
PLAT791_ALERT_4_G Model has Chirality at C3A (Chiral SPGR) S Verify
PLAT791_ALERT_4_G Model has Chirality at C3B (Chiral SPGR) S Verify
PLAT791_ALERT_4_G Model has Chirality at C4A (Chiral SPGR) S Verify
PLAT791_ALERT_4_G Model has Chirality at C4B (Chiral SPGR) S Verify
PLAT791_ALERT_4_G Model has Chirality at C7A (Chiral SPGR) R Verify
PLAT791_ALERT_4_G Model has Chirality at C7B (Chiral SPGR) R Verify
PLAT791_ALERT_4_G Model has Chirality at C8A (Chiral SPGR) R Verify

```
PLAT791_ALERT_4_G Model has Chirality at C8B         (Chiral SPGR)          R Verify
PLAT791_ALERT_4_G Model has Chirality at C9A         (Chiral SPGR)          R Verify
PLAT791_ALERT_4_G Model has Chirality at C10A        (Chiral SPGR)          S Verify
PLAT791_ALERT_4_G Model has Chirality at C11A        (Chiral SPGR)          R Verify

PLAT860_ALERT_3_G Number of Least-Squares Restraints .............          5 Note
```

```
   0 ALERT level A = Most likely a serious problem - resolve or explain
   0 ALERT level B = A potentially serious problem, consider carefully
   1 ALERT level C = Check. Ensure it is not caused by an omission or oversight
  27 ALERT level G = General information/check it is not something unexpected

   2 ALERT type 1 CIF construction/syntax error, inconsistent or missing data
   4 ALERT type 2 Indicator that the structure model may be wrong or deficient
   3 ALERT type 3 Indicator that the structure quality may be low
  19 ALERT type 4 Improvement, methodology, query or suggestion
   0 ALERT type 5 Informative message, check
```

It is advisable to attempt to resolve as many as possible of the alerts in all categories. Often the minor alerts point to easily fixed oversights, errors and omissions in your CIF or refinement strategy, so attention to these fine details can be worthwhile. In order to resolve some of the more serious problems it may be necessary to carry out additional measurements or structure refinements. However, the purpose of your study may justify the reported deviations and the more serious of these should normally be commented upon in the discussion or experimental section of a paper or in the "special_details" fields of the CIF. checkCIF was carefully designed to identify outliers and unusual parameters, but every test has its limitations and alerts that are not important in a particular case may appear. Conversely, the absence of alerts does not guarantee there are no aspects of the results needing attention. It is up to the individual to critically assess their own results and, if necessary, seek expert advice.

Publication of your CIF in IUCr journals

A basic structural check has been run on your CIF. These basic checks will be run on all CIFs submitted for publication in IUCr journals (*Acta Crystallographica*, *Journal of Applied Crystallography*, *Journal of Synchrotron Radiation*); however, if you intend to submit to *Acta Crystallographica Section C* or *E* or *IUCrData*, you should make sure that full publication checks are run on the final version of your CIF prior to submission.

Publication of your CIF in other journals

Please refer to the *Notes for Authors* of the relevant journal for any special instructions relating to CIF submission.

PLATON version of 03/05/2019; check.def file version of 29/04/2019

Datablock chde_mehle_p21 - ellipsoid plot

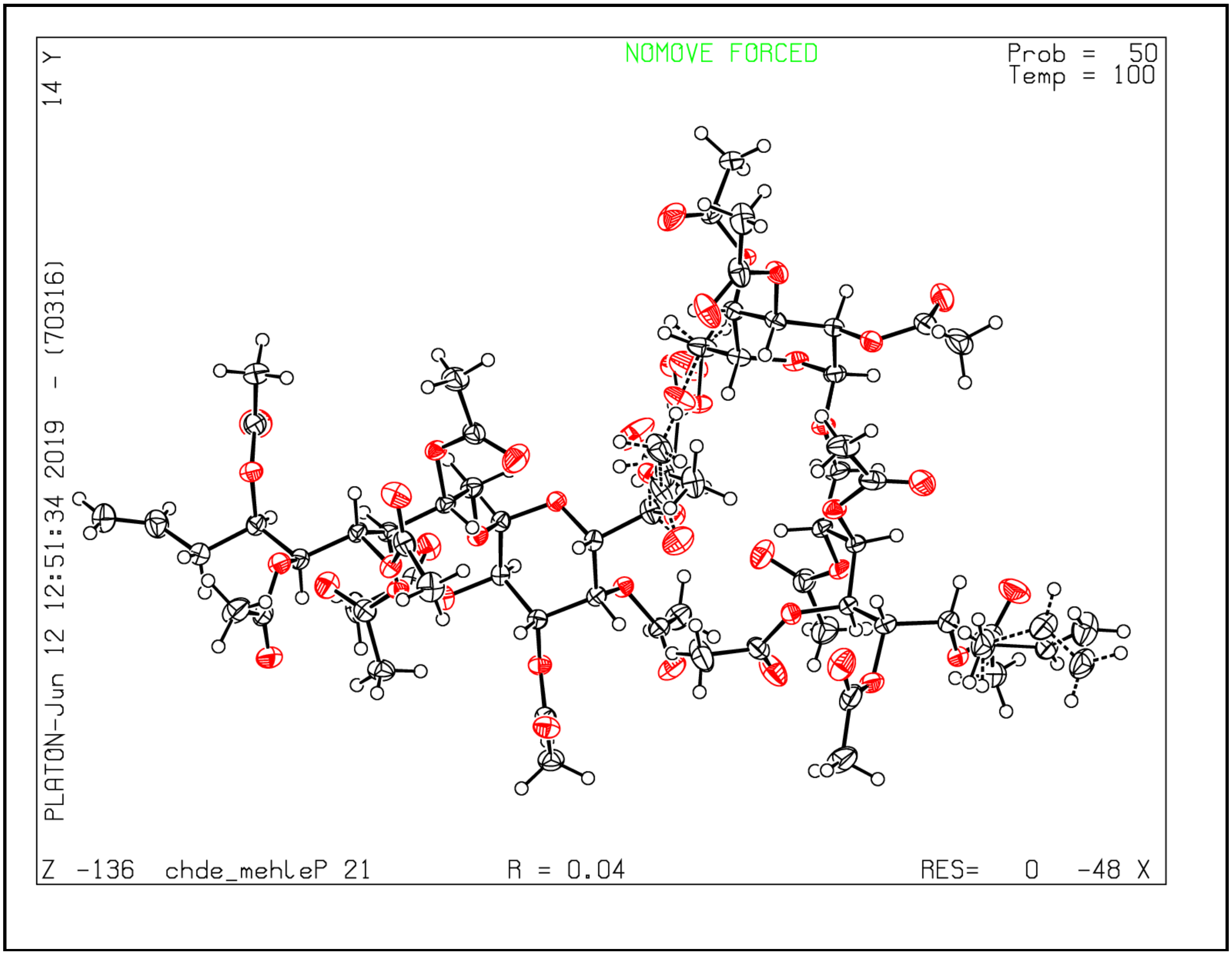

Download CIF editor (publCIF) from the IUCr
Download CIF editor (enCIFer) from the CCDC
Test a new CIF entry
